# Assessing prognosis of pulmonary embolism using tissue-Doppler echocardiography and brain natriuretic peptide

**DOI:** 10.1590/S1679-45082013000300013

**Published:** 2013

**Authors:** Ana Clara Tude Rodrigues, Adriana Cordovil, Cláudia Gianini Mônaco, Laise Antônia Bonfim Guimarães, Wércules Antônio Alves de Oliveira, Claudio Henrique Fischer, Edgar Bezerra de Lira, Marcelo Luiz Campos Vieira, Samira Saady Morhy

**Affiliations:** 1 Hospital Israelita Albert Einstein, São Paulo, SP, Brazil.

**Keywords:** Pulmonary embolism, Atrial natriuretic factor, Echocardiography Doppler/methods, Hypertension, pulmonary

## Abstract

**Objective:**

To assess prognosis of pulmonary thromboembolism using tissue Doppler echocardiography and brain natriuretic peptide.

**Methods:**

Patients aged over 18 years were evaluated within 24 hours of confirmed diagnosis (chest tomography/pulmonary scintigraphy) of pulmonary embolism using two-dimensional echocardiography and tissue Doppler for right ventricular systolic (s') velocities, strain, tissue tracking and myocardial performance index. Plasma brain natriuretic peptide was also obtained within 24 hour. The influence of echocardiographic and clinical variables on mortality was examined (up to 12 months) using Cox regression analysis.

**Results:**

Out of 118 patients, 100 patients were included in the study (60 males, aged 55±17 years). Right ventricular dysfunction was observed in 28% using two-dimensional echocardiography. Tissue Doppler right ventricular variables (s' velocities, tissue tracking and strain) were decreased only for patients with right ventricular dysfunction, whereas myocardial performance index and systolic pulmonary artery pressure were increased. Mean brain natriuretic peptide value was 66±111pg/mL, also increased in patients with right ventricular dysfunction (136±146pg/mL). Mortality was 11% and related to age, malignancy and brain natriuretic peptide levels. The only echocardiographic variables capable of predicting events by univariate analysis were pulmonary pressure and right ventricular s' velocity. However, multivariate analysis showed only malignancy to predict mortality in this group.

**Conclusion:**

Lower tissue Doppler systolic velocities and elevated brain natriuretic peptide levels are associated with poorer prognosis in patients with pulmonary thromboembolism; but only malignancy emerged as an independent predictor of mortality.

## RESUMO

**Objetivo:**

Avaliar o prognóstico do tromboembolismo pulmonar usando o ecocardiograma com Doppler tecidual e o peptídeo atrial natriurético.

**Métodos:**

Pacientes com idade acima de 18 anos foram avaliados pelo ecocardiograma bidimensional e Doppler tecidual para medidas das velocidades miocárdicas (s'), *strain* e índice de *performance* miocárdica do ventrículo direito até 24 horas da confirmação diagnóstica do tromboembolismo pulmonar (tomografia/ cintilografia), sendo também o peptídeo atrial natriurético obtido até 24 horas. A influência das variáveis na mortalidade até 1 ano foi testada pela regressão de Cox.

**Resultados:**

Dos 118 pacientes estudados, 100 foram incluídos, sendo 60 homens, com idade de 55±17 anos. Pelo ecocardiograma bidimensional, 28% dos pacientes apresentavam disfunção do ventrículo direito. As medidas da onda s', *strain* e deslocamento estiveram diminuídas para tais pacientes, que apresentavam, ainda, índice de *performance* miocárdica e pressão sistólica pulmonar aumentados. O peptídeo atrial natriurético médio foi de 66±111pg/mL, sendo 136±146pg/mL para pacientes com disfunção do ventrículo direito. A mortalidade foi 11% e pela análise univariada, relacionada à idade, neoplasia e peptídeo atrial natriurético. Entre as variáveis ecocardiográficas, somente a onda s' do Doppler tecidual e a pressão pulmonar associaram-se à maior mortalidade. Pela análise multivariada, entretanto, a presença de neoplasia foi o único preditor de óbito.

**Conclusão:**

Velocidades miocárdicas diminuídas e peptídeo atrial natriurético elevado estão associados a pior prognóstico em pacientes com tromboembolismo pulmonar, mas, nessa população, somente a presença de neoplasia foi capaz de predizer a mortalidade de maneira independente.

## INTRODUCTION

Mortality in pulmonary thromboembolism (PE) is essentially related to hemodynamic instability resulting from right ventricular dysfunction^(1–3)^, thus requiring appropriate evaluation of this chamber. Two-dimensional echocardiogram emerges as the preferred test to assess right ventricular (RV) performance since it is non-invasive, devoid of radiation, portable and can be serially undertaken. However, echocardiographic evaluation presents limitations because of RV structural complexity. Alternatively, tissue Doppler (TD) can be used to complement the two-dimensional echocardiogram^(4)^. This methodology relies on the analysis of myocardial velocities to increase the sensitivity of the examination to detect myocardial dysfunction^(5)^. In addition, TD can be used for prognostic evaluation in certain situations, such as identification of higher mortality and cardiovascular events in patients with cardiomyopathy and RV involvement^(6)^. However, there is no description of the influence of TD in the prognosis of patients with PE. On the other hand, in regard to the B-type atrial natriuretic peptide (BNP), although its importance for risk stratification is well-known for patients with heart failure, there is limited data regarding BNP and prognosis in PE^(7,8)^. Patients with PE and increased BNP seem to present a higher morbidity and mortality^(7)^, but with a lower cutoff value (BNP<50pg/mL) than that used for heart failure. However, there is no conclusive data about the contribution of this biomarker specifically in regard to mortality after PE^(8,9)^.

## OBJECTIVE

To prospectively evaluate right ventricular function using two-dimensional echocardiogram with tissue Doppler in patients with pulmonary thromboembolism, analyzing its relation with the B-type atrial natriuretic peptide and the prognosis.

## METHODS

### Patients

From August 2007 to January 2010, all patients admitted to the Emergency Room Unit or inpatients with clinical suspicion of PE (pain/dyspnea of sudden onset in the previous week) were invited to participate in the study. PE was confirmed by chest multi-slice computed tomography showing a complete or partial filling defect in the pulmonary vessels or by a pulmonary scintigraphy with a high probability of PE. Patients with left ventricular dysfunction (ejection fraction <55% by echocardiogram) were not included in the study to avoid the influence on BNP^(10)^. Other exclusion criteria were: chronic obstructive pulmonary disease, arrhythmias (atrial fibrillation or frequent premature beats), inadequate echocardiographic window and missing BNP measurements. All the patients were treated with anticoagulation (unfractionated heparin or low-molecular weight heparin, at the attending physician discretion); patients with hemodynamic instability (blood pressure <90/60mmHg and signs of poor peripheral perfusion) received thrombolytic therapy (tissue plasminogen activator, 100mg intravenously for 2 hours). Patients were prospectively evaluated during hospital stay and followed after discharge via telephone calls up to 12 months to assess mortality. The study was approved by the institutional review board (CAAE: 0064.0.028.000-07) and all the patients signed a written informed consent form to participate in the study.

### Echocardiography

Patients underwent a comprehensive echocardiogram (Vivid 7, GE Medical Systems, Horten, Norway) up to 24 hours after the event, including measurements of the left ventricular (LV) diameters and ejection fraction according to the recommendations of the American Society of Echocardiography^(11)^. Pulsed and continuous Doppler and color flow mapping were used to evaluate the presence and grade of valve regurgitation. Additional measurements of the ventricular diastolic diameters were obtained from the apical four-chamber view to obtain the RV/LV ratio. Parasternal, apical and subcostal views were used to evaluate RV systolic contractility. The presence of RV hypokinesia associated with abnormal septum motility (flattening) and/or RV dilation (RV/LV ≥1) was regarded as RV dysfunction by two-dimensional echocardiogram. Pulmonary pressure measurements were obtained from the tricuspid regurgitation, and the RV-right atrial gradient was added to the right atrial pressure estimated by the collapsibility of the inferior vena cava. Transmitral Doppler measurements were obtained for evaluation of the diastolic function (E and A waves, and E wave deceleration time).

### Tissue Doppler

**Figure 1:**
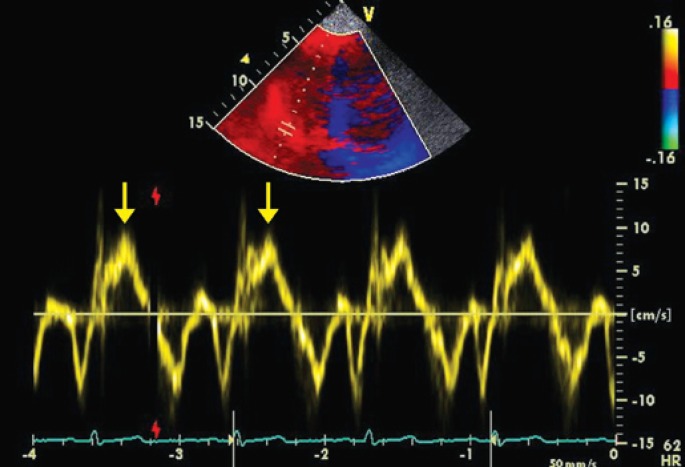
Tissue Doppler of the tricuspid annulus showing decreased (7cm/s) systolic velocity (s') in a patient with pulmonary thromboembolism and moderate right ventricular dysfunction (yellow arrows)

TD was obtained from the apical 4-chamber view of the septal, lateral and RV (tricuspid) anullus for measurements of systolic (s') and diastolic (e' and a') velocities, with the cursor parallel to the ventricular wall to minimize the influence of the angle on Doppler measurements (Figure 1). Estimation of the LV filling pressures was given by the E/e' ratio (average of septal and lateral walls). The presence of elevated LV filling pressures (E/e' >13) was regarded as diastolic dysfunction, since systolic function was preserved^(12)^. Patients were asked to breathe more slowly during the Doppler measurements. TD with color flow mapping was carried out from the apical 4-chamber view, with sector scan size and depth optimized to acquire images with a minimum frame rate of 100 fps, for adequate measurements of variables such as tissue tracking, strain and strain rate. Myocardial performance index (MPI) was obtained by TD of the RV and given as the ratio between isovolumetric contraction time (ICT) + isovolumetric relaxation time (IRT) divided by the ejection time (ET): MPIRV=(ICT+ IRT)/ET^(13)^. The average of three Doppler measurements was used for analysis. The data were digitally stored and subsequently analysed.

### Measurements of the B-type atrial natriuretic peptide

Plasma BNP levels were measured up to 24 hours after confirmed PE. Blood was collected in a tube containing ethylenediaminetetraacetic acid and separated by centrifugation. BNP was measured by radioimmunoassay (Advia Centaur assay^®^, Siemens Heathcare Diagnostics, Bayswater, Australia).

### Statistics

Continuous variables were expressed as mean±standard deviation (SD) while categorical variables were expressed in proportions. Univariate analysis was used to assess the influence of clinical and echocardiographic variables on mortality up to 12 months after PE; χ^2^ and Student's t test were used to test categorical and continuous variables, respectively. Multivariate Cox regression analysis was used to evaluate the influence of variables on the 12-month survival. Statistical analysis was performed with the program Statistical Package for the Social Science (SPSS), version 17.0 (SPSS, Inc, Chicago, IL). Statistical significance was determined at the 0.05 level. Measurements of TD myocardial velocities, tissue tracking, strain and strain rate were repeated after 3 months to evaluate inter-observer and intra-observer variability and tested by the intra-class correlation coefficient.

## RESULTS

**Figure 2:**
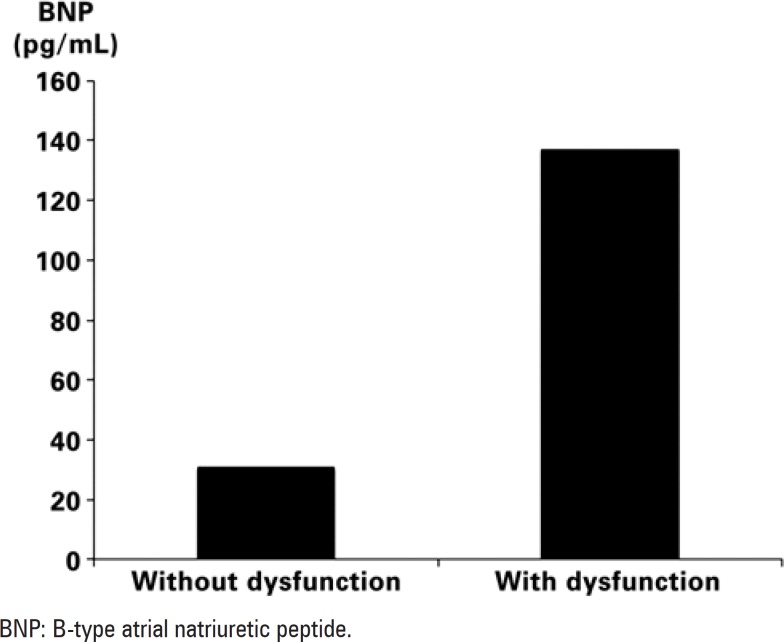
Values of atrial natriuretic peptide for patients with and without right ventricular dysfunction on two-dimensional echocardiogram

From a total of 118 patients with confirmed diagnosis of PE by tomography (n=114) or ventilation/pulmonary perfusion scintigraphy (n=4), 7 had inadequate echocardiographic windows, 5 refused to participate in the study, 2 did not have their BNP measured, 2 had associated left ventricular dysfunction and 2 presented arrhythmia. Therefore, the group included 100 patients and none of them was lost to follow-up. Deep venous thrombosis (29%) was the most frequent predisposing factor, followed by malignancy (24%), surgery (22%) and long-distance flight (6%). Most patients underwent conventional treatment, with only 4 patients treated with thrombolysis. Male gender (60%) was more predominant, with a mean age of 55±17 years. The mean BNP value found was 66±111pg/mL. Tricuspid regurgitation was present in 66 patients, and pulmonary pressure was 41±13mmHg. Of the 100 patients studied, 28 showed RV dysfunction according to the pre-established criteria: measurements of RV TD variables (s' velocity, strain and tissue tracking) were decreased only for patients with RV dysfunction; MPI and pulmonary artery systolic pressure were also increased for these patients. BNP, in turn, was 30±39pg/mL (median=12pg/mL) for patients without RV dysfunction (Figure 2), and increased in patients with echocardiographic RV dysfunction (136±146pg/mL, median=64pg/mL). Mortality was 11% and, by univariate analysis, and related to age, malignancy and BNP values (both absolute value and BNP >50pg/mL). As pre-established, none of the patients presented with left ventricular systolic dysfunction; in regard to diastolic function, average LV filling pressures (estimated by E/e') was within previously described normal limits, and it was similar for patients with and without RV dysfunction. The echocardiographic variables studied are described on chart 1.

**Chart 1:** Data of patients with pulmonary thromboembolism

LVEF (%)	67±6
RV s' (cm/s)	12.3±3.5
Tracking (mm)	16.9±5.2
RV strain (%)	22±9.3
RV/LV	0.9±0.3
PASP (mmHg)	41±13
MPI	0.51±25
E/e'	8.2±2.8
BMI (kg/m2)	26.4±1.6
HR (bpm)	80.8±16.3

LVEF: left ventricular ejection fraction; RV s': right ventricular systolic myocardial velocity; RV: right ventricle; RV/LV: right/left ventricular ratio (apical view); PASP: pulmonary artery systolic pressure; MPI: myocardial performance index; E/e': transmitral initial diastolic velocity (E)/average of septal and lateral initial diastolic velocity(e') ratio; BMI: body mass index; HR: heart rate.

### Evaluation of events

During the follow-up period of 12 months there were 11 deaths; only 3 occurred within 1 month of PE. According to the univariate analysis, mortality was related to plasma BNP (both absolute values and BNP >50pg/mL), age and the presence of malignancy. Of the echocardiographic variables, only TD RV s' velocity and pulmonary systolic pressure were correlated with mortality. No other variable derived from TD, or even MPI, showed significant influence on survival (Table 1). Multivariate analysis, however, showed that only the presence of malignancy was independently associated with mortality in this group of patients (Table 2).

**Table 1:** Predictors of mortality by Cox regression model

	Relative risk	CI 95%	p value
Age	1.05	1.01-1.09	0.009
Gender (female)	0.9	0.28-2.87	0.9
DVT (yes)	0.5	0.11-2.33	0.3
Surgery (yes)	0.7	0.15-3.14	0.6
Neoplasm (yes)	20.2	4.41-93.1	<0.001
Hypotension/shock	3.3	0.42- 25	0.2
BNP	1.0	1.00 −1.01	0.04
RV Dysfunction	2.5	0.44-4.90	0.5
PASP	1.1	1.02-1.12	0.004
RV s'	0.8	0.67-0.97	0.02
RV/LV	4.5	0.56 −35.7	0.2
Tracking	1.0	0.85-1.09	0.5
RV strain	1.0	0.92-1.07	0.8
Septal E/e'	1.1	0.89-1.36	0.2
MPI of RV	0.5	0.04-6.57	0.6

CI 95%: 95% confidence interval; DVT: deep venous thrombosis; BNP: B-type natriuretic peptide; PASP: pulmonary artery systolic pressure; RV s': right ventricular systolic myocardial velocity; RV/LV: right/left ventricular ratio (apical view); E/e': transmitral initial diastolic velocity (E)/average of septal and lateral initial diastolic velocity(e') ratio; MPI: myocardial performance index.

**Table 2:** Predictors of mortality by multivariate analysis, Cox regression

	Relative risk	CI 95%	p value
Age	1.1	0.99-1.23	0.08
Neoplasm (yes)	8.6	1.4-52.8	0.001
BNP	1.3	0.88 −1.95	0.2
BNP >50	5.3	0.99-9.90	0.2
PASP	1.04	0.97-1.12	0.3
RV s'	0.8	0.67-0.97	0.2

CI: 95% confidence interval; BNP: B-type natriuretic peptide; PSAP: pulmonary artery systolic pressure; RV s': right ventricular systolic myocardial velocity.

### Inter-observer and intra-observer variation

Intraclass correlation coefficient was 0.98 (intra-observer) and 0.97 (inter-observer) for RV s' wave velocity; 0.98 (intra-observer) and 0.96 (inter-observer) for tissue tracking and 0.95 (intra-observer) and 0.94 (interobserver) for strain measurements. Measurements of strain rate were not used for the analysis because of their marked variability.

## DISCUSSION

Acute PE is a disease with high prevalence, frequently underdiagnosed, with resulting treatment failure and complications. Prognosis of PE is related to the preexisting cardiovascular disease, the degree of pulmonary hypertension and vascular obstruction, and mainly, to the presence of RV dysfunction. Two-dimensional echocardiogram is routinely used to evaluate RV performance in PE in order to identify patients with higher risk of morbimortality, with indication of a more aggressive treatment^(2,3)^. Our prevalence of RV dysfunction was relatively low (28%) compared to that in literature^(1,3)^. Two factors may have contributed to this finding: first, since patients with left ventricular dysfunction were excluded, those patients with preexisting RV myocardial involvement associated with cardiomyopathy may have been equally eliminated. Additionally, in other reports, multiple echocardiographic criteria such as RV dilatation and hypokinesia, the presence of pulmonary hypertension, inferior vena cava dilatation and RV free wall hypertrophy have been used to evaluate the RV. These criteria, used either isolated or in combination might lead to a higher sensibility (and, consequently, lower specificity) of the method. To characterize RV dysfunction in our study we did not include the presence of pulmonary hypertension, dilation of inferior vena cava or hypertrophy of the chamber.

When adequately treated, mortality in PE is low (<5%). Patients who are hemodynamically unstable present a higher risk because of RV dysfunction, with mortality higher than 25%^(1,2)^. In our population, consisting mostly of hemodynamically stable patients, mortality up to 1 year was 11%. Isolated RV dysfunction evaluated by the two-dimensional echocardiogram, however, was not able to identify a group of patients with higher mortality. Despite the established risk for patients with PTE and hemodynamic instability due to right ventricular involvement, a systematic review of the studies with PE in hemodynamically stable patients did not confirm the utility of two-dimensional echocardiogram to predict a higher risk of death^(14)^. The power of echocardiographic RV dysfunction to predict inhospital mortality was low (65% sensibility, positive predictive value of 4%)^(15)^. This is important because, although there is consensus in regard to more aggressive treatment in the presence of hemodynamic instability, some institutions may turn the same treatment to RV echocardiographic dysfunction, increasing the risks of thrombolysis. On the other hand, differently from the two-dimensional echocardiogram, TD RV myocardial velocities were able to identify the patients with a higher risk of death up to 1 year after the event. TD has been shown to be a very useful technique for quantitative evaluation of LV diastolic and systolic functions, being more sensitive to detect earlier functional myocardial abnormalities^(16,17)^. In recent studies involving other diseases (including heart failure and hypertension), myocardial systolic velocities were able to predict mortality or the presence of cardiovascular events^(18)^. TD velocities have been proved to be a rapid and simple means to study RV function and prognosis in situations where this chamber is observed to be impaired^(4,19)^, however, there are no studies relating TD with prognosis in PE. In our study though myocardial velocities were more sensitive to assess outcome, they were not able to independently predict death after PE. TD is influenced by age, with decreased velocities in elderly patients, which may have contributed to the lower accuracy of this method. Other variables derived from TD (strain and tissue tracking) were not able to predict events even in univariate analysis.

In regard to the MPI, it was observed that this measurement was increased in patients with RV dysfunction; however, no midterm correlation with prognosis after PE was observed in this population. Increased pulmonary resistance, such as seen in PE, would lead to a prolonged RV isovolumetric time and, consequently, to an increased RV MPI. Measurements of PMI by TD can be technically difficult in patients with dysfunction, which may partially explain this finding.

In regard to BNP, it was elevated in patients with RV dysfunction, and the association with mortality in the univariate analysis confirmed the literature findings^(8)^. Pulmonary vasoconstriction after PE results in increased right heart work to increase pulmonary pressure; depending on the pulmonary pressure increase, RV failure may ensue. Since BNP is secreted in response to increased atrial or ventricular stress, it reflects pressure or volume overload^(20)^. Pieralli et al.^(8)^ studied patients with PE who were hemodynamically stable. A BNP value >50pg/mL, along with echocardiogram, identified a group of patients with a higher number of adverse complications (death, use of mechanical ventilation or necessity of embolectomy). In the present study population, we assessed the association of BNP with mortality, not with complications, and statistical significance was not maintained after the multivariate analysis. BNP relation with RV pressures is not linear, and several factors influence secretion and elimination of this biomarker, including left ventricular systolic dysfunction with increased filling pressures. To avoid this influence on BNP, patients with systolic ventricular dysfunction were excluded. Previous studies where BNP was presented as an independent factor for higher risk of events in PE, either lack reference to left ventricular function^(8)^ or include patients with heart failure^(9)^, which would certainly have an influence on BNP levels. In the absence of left ventricular dysfunction, the association of BNP with mortality in PE is probably limited, and its importance should be carefully evaluated. Diastolic dysfunction is only modestly correlated with BNP, with better correlations in cases of marked diastolic dysfunction or in the setting of associated systolic dysfunction^(21,22)^. In this study, since it would not be possible to exclude patients with diastolic dysfunction^(23)^, LV filling pressures were estimated. The low E/e' values found suggest the absence of marked diastolic dysfunction; we believe that the influence of diastolic dysfunction on BNP values can thus be minimized.

Other factors associated with higher mortality were increased pulmonary pressures and older age. Persistently elevated pulmonary pressures are associated with a higher prevalence of chronic PE and RV dysfunction^(24)^. Older age is an additional factor for higher comorbidity by other diseases.

Regarding PE association with malignancy, venous and arterial thromboembolism are frequently found in patients with cancer; they can be potential causes of morbimortality and are predictors of a higher risk in this population^(25,26)^. Patients with PE and malignancy more frequently have advanced disease and have a worse prognosis^(27)^. In our population, a large number of patients with PE presented with malignancy (24% of the studied population), with an inevitable influence on mortality, especially due to the low prevalence of hypotension or shock in the group: only one death was directly attributed to massive PE with hemodynamic instability and right ventricular failure. Of the 11 deaths, 10 patients had cancer. Mortality was related to the baseline disease in all the patients, except for one patient (infection). In the midterm follow-up, PE was most likely an important contributor to mortality than the cause of death itseft. However, it is important to emphasize that, differently from other studies, most patients were hemodynamically stable and that patients with heart failure were excluded.

Some limitations to the study should be pointed out: only the basal segment of RV was analyzed by TD. However, it is known that RV involvement in PE affects the entire free wall, occasionally sparing the apex^(28)^, thus allowing adequate analysis by TD. Additionally, in regard to the technique, TD depends on image quality, requiring a high frame rate for an adequate tracings. Although pulsed TD has shown to be of excellent quality, probably as a result of interference related to the respiratory pattern, other TD measurements (strain rate) may have suffered more influence from image quality.

## CONCLUSION

The presence of malignancy is related to higher mortality in patients with acute pulmonary thromboembolism. In the studied population, tissue Doppler and B-type atrial natriuretic peptide did not correlate independently with a worse prognosis.
